# A Chronic Glottic Foreign Body Diagnosed by Radiograph after 9 Months of Symptoms

**DOI:** 10.1155/2018/4718428

**Published:** 2018-05-23

**Authors:** Laura H. Swibel Rosenthal, Virginia Smith-Bronstein, Santino Cervantes, James W. Schroeder

**Affiliations:** ^1^Division of Pediatric Otolaryngology, Ann and Robert H. Lurie Children's Hospital of Chicago, Chicago, IL, USA; ^2^Northwestern University Feinberg School of Medicine, Chicago, IL, USA

## Abstract

A six-year-old girl presented to an emergency room after describing choking on a rubber band. She was in no distress and was discharged. Over the course of the next 9 months, she had numerous outpatient and emergency room visits due to intermittent stridor, difficulty breathing, and hoarseness. Eventually, dedicated airway films revealed a laryngeal foreign body. During rigid bronchoscopy, a two-centimeter rubber band was discovered in the larynx. It extended from the supraglottis, through the glottis, and into the subglottis. It was successfully removed. The patient was asymptomatic 24 hours later. This case highlights the appropriate evaluation and management of a child with stridor.

## 1. Introduction

Laryngeal foreign bodies are rarely reported in the literature and have not been studied in large numbers. A laryngeal foreign body typically presents with acute airway obstruction. If the foreign body is not quickly expelled with a cough or aspirated deeper into the lower airway, it will result in death. Therefore, a chronic laryngeal foreign body is very rare. Chronic nasal, tracheal, and bronchial airway foreign bodies are much more common as they are less likely to be immediately fatal. In a 10-year review of 135 cases of foreign bodies in the airway, there were 2 cases (1.4%) of a laryngeal foreign body. One was a hotdog at the laryngeal inlet, and the patient died from respiratory arrest despite initiation of the Heimlich maneuver and cardiopulmonary resuscitation (CPR). The other patient survived after having a seashell removed from the larynx that was originally identified using an airway film (the timing of which was not reported) [1].

The presentation of any airway foreign body, regardless of location, varies widely, and therefore, the diagnosis can be evasive. Patients may have acute total airway obstruction or chronic respiratory symptoms, or they may be relatively asymptomatic for a period of time. In 2001, there were 17,537 visits to the emergency department (ED) in the United States for nonfatal, choking-related episodes in children less than 14 years [2]. The prior year, 160 children died from airway obstruction from an aspirated body [2]. Although prevention is one essential aspect of this epidemic, proper management of these patients in the ED, outpatient clinic, and operating room can significantly reduce morbidity and mortality.

A partially obstructive laryngeal foreign body will cause cough, hoarse voice, and biphasic stridor. In a meta-analysis, the most common symptoms (and pooled proportions) of an airway foreign body, regardless of location, were cough (0.612), choking (0.468), dyspnea (0.346), throat pain (0.290), fever (0.187), thoracic pain (0.140), nonspecific symptoms (0.098), no symptoms (0.079), vomiting (0.074), hoarseness (0.048), blood-stained mucous (0.021), and unconsciousness (0.008) [3]. In the same meta-analysis, physical examination revealed decreased air entry by visualization or auscultation (0.454–0.659), tachypnea (0.476), acute respiratory distress (0.378), abnormal breath sounds of wheezing, rhonchi, crackles, and rales (0.126–0.334), accessory muscle use (0.196), purulent discharge (0.189), stridor (0.177), and other findings [3]. As these symptoms and physical findings are nonspecific and common in other very common illnesses, a delay in diagnosis is possible. A delay in diagnosis of an airway foreign body is attributed to lack of a history to suggest a foreign body, false negative findings on imaging, and lack of imaging [1]. A delay in diagnosis would be expected to occur more with radiolucent objects than with radiopaque foreign bodies. A delayed diagnosis is relatively common. Boufersaoui et al. conducted a retrospective study of 2624 foreign body aspiration cases and found that the delay between aspiration and removal was 2 to 8 days in 66% of cases, while only 9% underwent extraction within 24 hours. Less than 25% of cases had foreign body removal that occurred greater than 8 days past aspiration event in their study population [4]. Tan et al. report a range of aspiration to removal extending to 3 years out from the aspiration event, with 6.7% removed in 2–12 months [1].

There are no published reports that specifically study the presentation of or the frequency of timely diagnosis and definitive treatment for isolated laryngeal foreign bodies. However, hoarseness is more commonly seen in patients with laryngeal foreign bodies as opposed to tracheal foreign bodies [1]. In this report, we present a unique case of a chronic radiopaque laryngeal foreign body and its management from workup to retrieval.

## 2. Case Report

**Figure 1:**
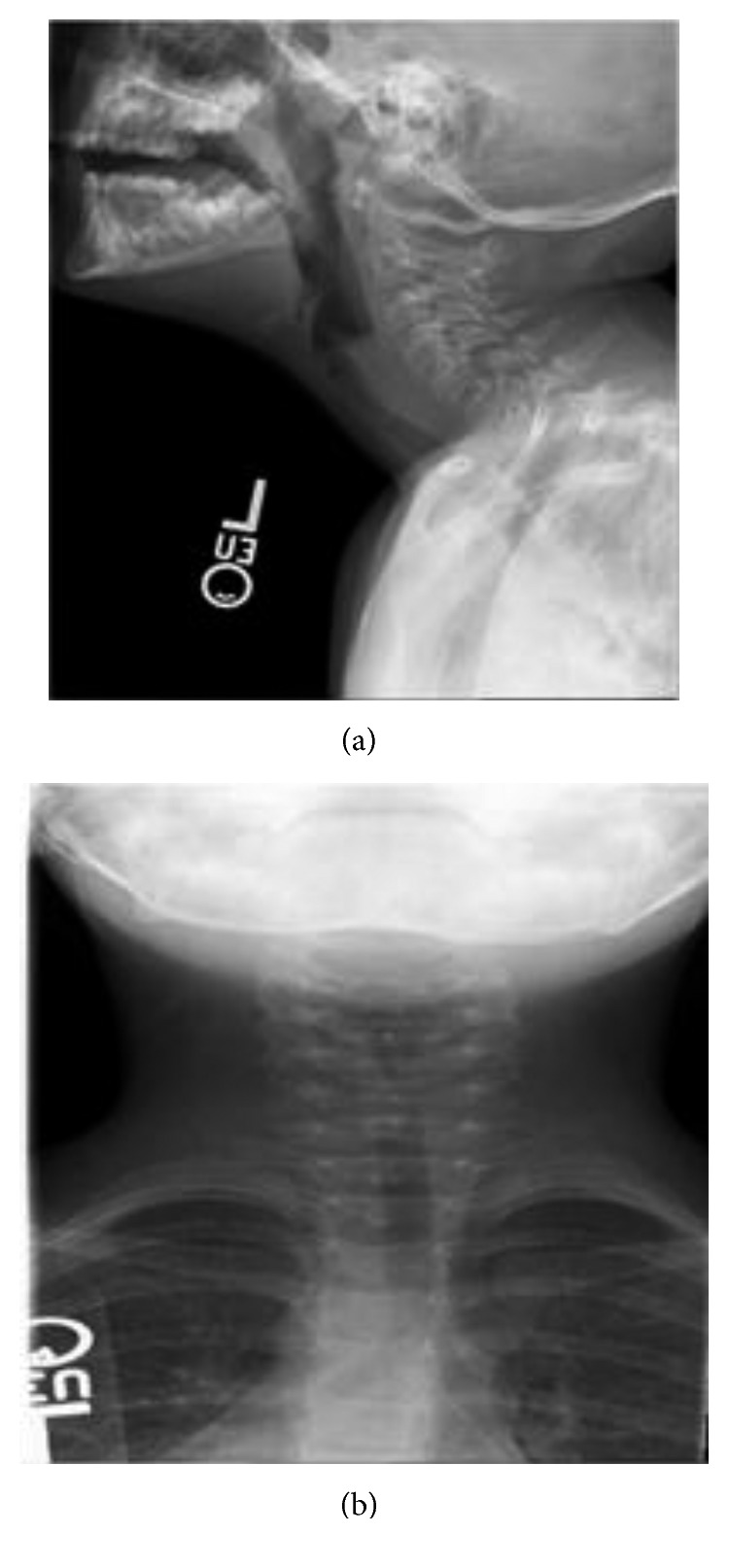
(a) Lateral airway film demonstrating a circular foreign body consistent with a rubber band spanning the glottis. (b) Anterior airway film demonstrating narrowing of the airway. The films suggest that soft tissue is encompassing the foreign body.

A healthy six-year-old female was transferred to the pediatric intensive care unit at the Ann and Robert Lurie Children's Hospital of Chicago at Northwestern University (a quaternary care institution) for a persistent barking cough and increased respiratory distress. Earlier that day, she had presented to a local immediate care facility due to a cough that had worsened over the prior two days. She was admitted to the hospital at the immediate care facility due to wheezing and shortness of breath that continued to worsen despite nebulized albuterol, nebulized racemic epinephrine, and subcutaneous epinephrine. Intravenous (IV) ampicillin/sulbactam was also given. The decision was made to transfer the child to a quaternary care facility for further workup and treatment. While in route, an anterior-posterior (AP) radiograph and a lateral chest radiograph (CXR), which had been taken prior to transportation, was reviewed and it revealed a circular foreign body extending from C3 to C6 (Figures 1(a) and 1(b)). This was the patient's first documented radiograph. Upon arrival, the patient had her first evaluation by a pediatric otolaryngologist.

The patient's parents reported that the patient had a barking cough and hoarseness that had varied in severity over the last 9 months. She had been diagnosed with recurrent croup. Upon further questioning, the family revealed that the child had a choking event while playing with a rubber band 9 months earlier. She was evaluated in a local emergency department shortly after the choking event and had had 4 to 5 emergency room and/or primary care physician visits over the last 9 months due to the barking cough and hoarse voice. She had received dexamethasone and oral steroids three times over the course of 9 months. This did often provide temporary relief of her symptoms. She did not have a direct or an indirect laryngeal exam, and no radiographs were taken.

Upon arrival at the quaternary institution, the patient was in mild distress. Her oxygen saturation was 97% on room air. She had a persistent biphasic stridor, hoarseness, and moderate tracheal and intercostal retractions. The CXR obtained at the local hospital did not adequately define the laryngeal structures. Repeat dedicated AP and lateral airway radiographs were obtained in order to better assess the airway and the presumed laryngeal foreign body. She was treated with an additional dose of nebulized racemic epinephrine and was given IV dexamethasone (0.5 mg/kg) upon arrival. The repeat airway films clearly demonstrated a laryngeal foreign body that was extending from the supraglottis into the subglottis. The patient was stable and cooperative, so a flexible fiber optic laryngoscopy (FFL) was performed through her nose. A loop of a rubber band was clearly visible in the supraglottis and extending through the glottis in an anterior-posterior orientation with one arm in the anterior commissure. There was granulation tissue in the anterior and posterior glottises. The vocal cords were mobile. The decision to perform bedside laryngoscopy in this case is controversial. The team felt strongly that this would assist with surgical planning. However, the airway films likely provided sufficient information to proceed without the additional laryngoscopy. Laryngoscopy in an uncontrolled setting may convert this partial laryngeal obstruction into a complete obstruction. In this case, the patient tolerated the procedure well and without an incident. The patient was then taken immediately to the operating room for microsuspension laryngoscopy and bronchoscopy.

The surgeons anticipated that the glottic and subglottic granulation tissue may cause bleeding or airway obstruction. The room and staff were prepared for an emergent tracheostomy in the event of total airway obstruction. There was a preoperative huddle that included the pediatric otolaryngologist, pediatric anesthesiologist, residents, fellows, and room staff to review the airway plan. The patient remained spontaneously ventilating while under general anesthesia. Mask ventilation with an oral airway was used when needed, but positive pressure ventilation was avoided, when possible, and a bronchoscope was available if needed for foreign body retrieval and/or ventilation. The patient's larynx was coated with topical 4% administered using an atomizer.

**Figure 2:**
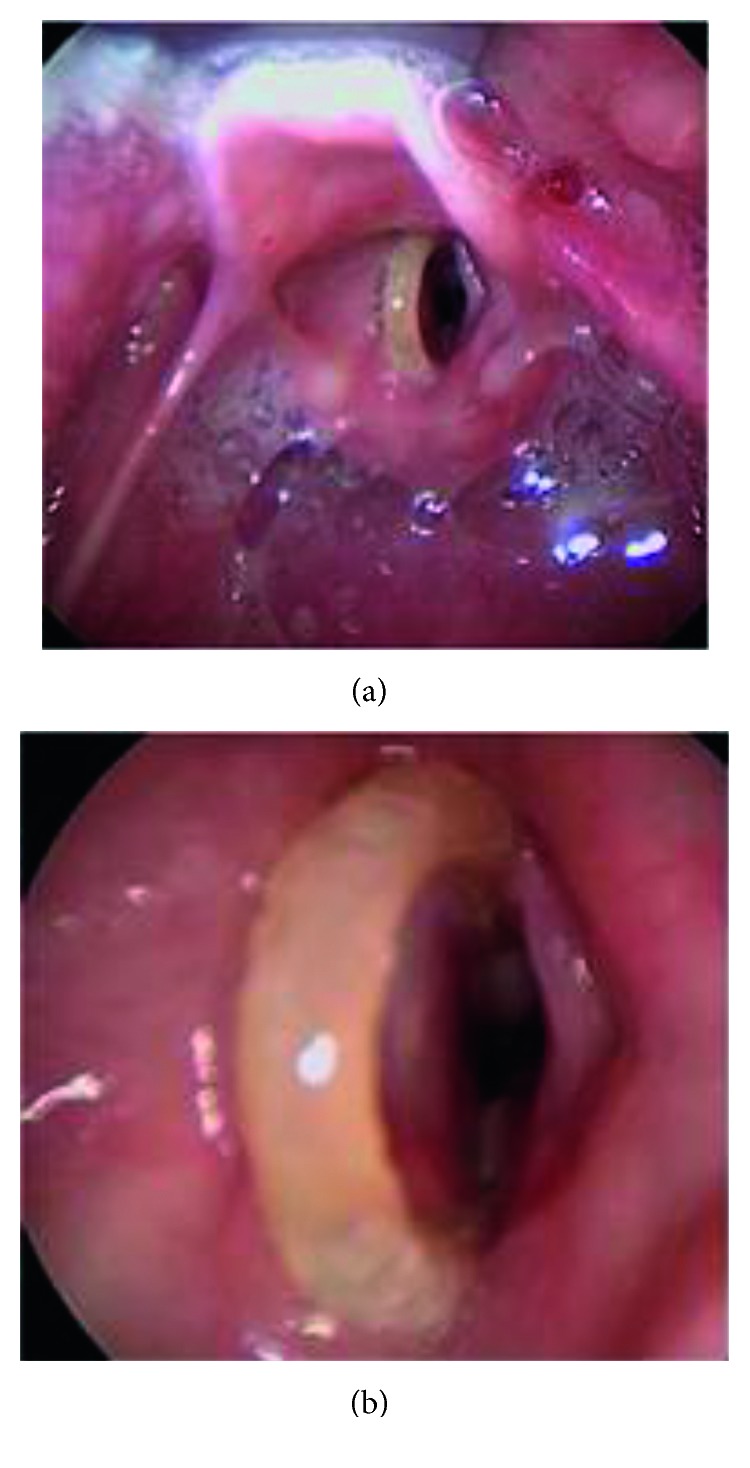
(a) Initial microlaryngoscopic view of the superior aspect of the rubber band overlying the left true vocal fold. (b) The foreign body spans the glottis entering the subglottis, where granulation tissue is encapsulating the object anteriorly and posteriorly.

**Figure 3:**
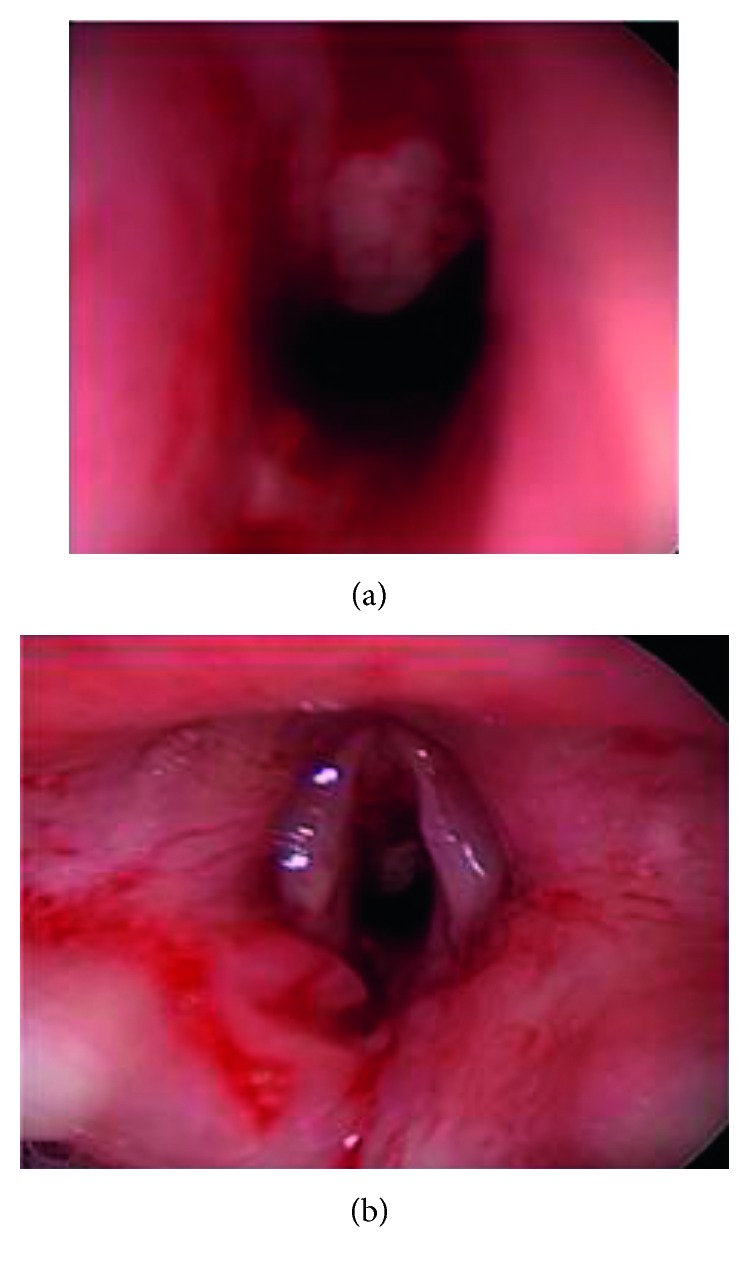
(a) Microlaryngoscopic view of the glottis immediately following foreign body removal. (b) There is significant airway stenosis secondary to the granulation tissue, which was subsequently removed.

Direct laryngoscopy was performed, and the foreign body was seen extending just above the vocal folds and in the interarytenoid space. A bronchoscopy was performed with photo documentation (Figures 2 and 3). A lumen was identified. Laryngoscopy was again used to expose the glottis and the two-centimeter rubber band in the glottis. The rubber band was engulfed in granulation tissue, which was anchoring the foreign body. It was removed under direct visualization with alligator forceps. Microbronchoscopy was then performed. Obstructive granulation tissue which had narrowed the airway was removed with the optical cup forceps. The anterior glottis and subglottis were most impacted by the granulation tissue. Granulation tissue was present in the intra-arytenoid space as well. Bleeding was controlled with topical oxymetazoline. The patient was awakened in the operating room and was transferred to the intensive care unit (ICU) where she was administered IV dexamethasone 0.25 mg/kg every 8 hours for 3 doses. She had no postoperative stridor, no respiratory distress, or increased work of breathing. She did not require racemic epinephrine, which was ordered as needed. Twenty-four hours later, the patient's hoarseness resolved. She remained completely asymptomatic. She was discharged without any medications. There was a plan for a repeat bronchoscopy one month later to assess granulation tissue and anterior glottis scar formation, but the patient did not follow-up. A phone call to the family approximately 3 months after the surgery confirmed that the patient was still asymptomatic.

## 3. Discussion

Primary care providers and otolaryngologists should be suspicious of an airway foreign body when a child presents with acute onset respiratory symptoms such as cough, noisy breathing, and respiratory distress, particularly following a choking episode. The patient presented in this case highlights the value of imaging, which was not performed until the patient had symptoms for 9 months despite persistent respiratory symptoms. It is possible that the patient presented to various immediate care settings with croup-like symptoms, rather than a regular primary care provider. Perhaps, the lack of care continuity in this case played a role in misdiagnosis of recurrent croup. In the meta-analysis of patients with airway foreign bodies, approximately 99% of patients had some types of radiograph, with findings that varied from normal (0.474) to positive for a radiopaque foreign body (0.246) to emphysema, atelectasis, pneumonia, pneumothorax, and others [3]. A laryngeal foreign body is rare, but given the patient's recurrent respiratory symptoms in this case, imaging should have been obtained sooner. Reasons for the delay in diagnosis of foreign bodies are physician-related in 17% of cases (for misdiagnosis of asthma, URI, or pneumonia), parent-related in 15% of cases (e.g., if care was not sought after a choking episode), or attributed to a negative history in 12% of cases (no suggestion of a foreign body or choking episode) [1]. It is possible that the patient or family only referred to the choking episode at the first emergency department visit 9 months before. In addition to the potential delay in diagnosis for a foreign body location that would typically present more acutely, this case highlights other unique aspects in the management of a laryngeal foreign body including (1) imaging options, (2) endoscopic options, and (3) safe operative and postoperative strategies.

When considering imaging for a potential foreign body, airway and chest radiography is often the first line in workup of a suspected airway foreign body, but it has a low sensitivity and specificity [5]. Chest radiographs are usually obtained first and positive 75.5% of the time for some findings (including emphysema, atelectasis, pneumonia, pneumothorax, foreign body, and others) [1]. Airway fluoroscopy can be a useful additional diagnostic tool, either as a routine initially or only if a CXR is negative or nonspecific [1, 6]. Computed tomography (CT) has a potential role in workup as it has a sensitivity profile that is better and may be more readily available than bronchoscopy [6], which could be the next step for the patient suspicious of having a foreign body. If a patient requires general anesthesia for a CT scan, then this may not be a good option.

Proper workup of respiratory symptoms often includes imaging, but one could also argue that the decision of whether or not to perform a laryngoscopy and a bronchoscopy for a suspected foreign body should be made primarily on history and physical examination. This is especially true if imaging is negative for a radiopaque foreign body. The decision to proceed with endoscopy should be made based on the patients symptoms [7]. This does not mean that imaging studies should be avoided. Alternatively, a patient with recurrent croup and chronic dysphonia would benefit from otolaryngology consultation and direct visualization of the larynx with FFL. Access to otolaryngology consultation and FFL may alter medical management in the ED or other settings.

Cutrone et al. have suggested flexible bronchoscopy as an additional option for workup when suspicion of a foreign body is low. The bronchoscopy could naturally include nasal and laryngeal examination by endoscopy prior to reaching the trachea and would be performed with intravenous sedation and topical anesthesia. In the same setting, the provider could convert towards rigid bronchoscopy, if a foreign body is identified [8]. This is not without risk, cost, and access issues. Direct laryngoscopy and bronchoscopy are similarly not without risk, cost, and access issues, yet they are imperative if a foreign body is present. The foreign body must be removed. Furthermore, there is a high likelihood of significant morbidity if a foreign body is left in the airway.

When taking an airway foreign body patient to the operating room, especially in the setting of a chronic foreign body, significant granulation tissue may make the case challenging. There may be airway obstruction or bleeding, which should be anticipated upon induction and during removal. Surgeons and anesthesiologists must be prepared for spontaneous respirations, intubation, or an emergent surgical airway. In one study, tracheostomy was performed in 4 of 342 cases of foreign body to provide to assist with retrieval and/or secure the airway. Indications for tracheostomy were chronic subglottic foreign bodies, sharp subglottic foreign bodies, or foreign bodies that were larger than the subglottic opening [9]. In addition to granulation tissue, other complications of a chronic airway foreign body may include stenosis, pulmonary infiltrates, infection, or tracheoesophageal fistula [6, 10]. Postoperative management and follow-up care for patients with chronic foreign bodies should be provided with these complications in mind. Izadi et al. reported the case of a 23-year-old woman with a large chicken bone in the larynx for 2 months. It was confused with the thyroid cartilage on the radiograph, and intraoperatively, there was significant granulation tissue. In this case, mitomycin was applied.

The literature on laryngeal airway foreign bodies is lacking in part because of their low incidence; therefore, a case report can be helpful in guiding management. Further studies are needed to apply evidence-based medicine to laryngeal foreign bodies. However, the similarities between laryngeal foreign bodies and airway foreign bodies in other locations such as the bronchi or even the nasal cavity can be extrapolated to improve our management. Guidelines for when to obtain imaging, when to perform an endoscopy, or how to manage the complications of a chronic laryngeal foreign body should be similar to those of other airway foreign bodies. However, there are many unique qualities of a chronic laryngeal foreign body that can be predicted. Symptoms of a laryngeal foreign body should be similar to those of any other chronic laryngeal pathology and include stridor, shortness of breath, cough, and dysphonia. As with the most pediatric foreign bodies from the ears to the esophagus, a provider must always be suspicious that local symptoms could be the result of a retained foreign body.

## 4. Conclusion

Glottic foreign bodies are an airway emergency. A choking episode followed by hoarseness, stridor, and difficulty breathing are typical presenting symptoms. We present a case of misdiagnosis of a glottic foreign body in a child. This case highlights the importance of a thorough history, exam, and appropriate imaging when evaluating a child with stridor. At a minimum, a chest radiograph that includes the larynx should be obtained. When the CXR is equivocal or does not demonstrate an obvious foreign body but the symptoms persist, dedicated airway films and/or laryngoscopy and bronchoscopy are warranted.
